# Perceptions of obesity in a UK leisure-based population of horse owners

**DOI:** 10.1186/1751-0147-57-S1-O6

**Published:** 2015-09-25

**Authors:** Philippa Morrison, Patricia Harris, Charlotte Maltin, Dai Grove-White, Caroline Argo, Clare Barfoot

**Affiliations:** 1 Department of Obesity and Endocrinology, University of Liverpool, Liverpool, UK; 2 Equine Studies Group, WALTHAM Centre for Pet Nutrition, Waltham on the Wolds, Leicestershire, UK; 3 School of Veterinary Medicine, University of Surrey, Guildford, Surrey, UK; 4 Mars Horsecare UK Ltd., Milton Keynes, Buckinghamshire, UK

## Introduction

Reduced workloads, improved husbandry and increased access to energy dense forage and concentrate feeds have contributed to the high prevalence of obesity among leisure horse populations. Obesity prevalence can also be linked to our ability to recognize and manage it accordingly. Currently, owner-perception of equine obesity is poorly understood.

## Objective

To evaluate owner-perceptions of equine obesity within the leisure horse sector.

## Methods

Internet-based survey was distributed through UK-based equine forums. A panel of 12 lateral-images of horses and ponies were presented.

## Tasks included

Asserting their involvement in the sector; identifying overweight animals; scoring the suitability of animals for participation in different equestrian activities on the basis of body weight.

## Results

Of 546 respondents, 98% were female. Amateur owners exceeded professional respondents (81%:19%). Key findings included; that only 11% correctly identified all overweight animals (6/12), but between 37 and 98% correctly identified individual overweight animals. Professional status did not influence an owner's ability to identify overweight animals. On assessing the weight/condition suitability of a sport horse, a cob and a pony for different disciplines, being overweight was considered more appropriate when animals were intended to be used for showing (p< 0.01).

## Conclusions

The study provided evidence that owner's may be less able to correctly identify overweight animals by visual appearance alone. Data support our anecdotal understanding that owners consider it appropriate that horses and ponies should carry more weight when competing in showing classes. These data will aid in targeting nutritional advice for horse owners.

